# Gadolinium decreases inflammation related to myocardial ischemia and reperfusion injury

**DOI:** 10.1186/1476-9255-6-34

**Published:** 2009-12-10

**Authors:** Jennifer L Strande, Kasi V Routhu, Anna Hsu, Alfred C Nicolosi, John E Baker

**Affiliations:** 1 Division of Cardiovascular Medicine, Medical College of Wisconsin, Milwaukee, Wisconsin, USA; 2 Department of Pharmacology and Toxicology, Medical College of Wisconsin, Milwaukee, Wisconsin, USA; 3 Division of Cardiothoracic Surgery, Medical College of Wisconsin, Milwaukee, Wisconsin, USA; 4 Department of Pharmacology and Toxicology, Medical College of Wisconsin, Milwaukee, Wisconsin, USA

## Abstract

**Background:**

The lanthanide cation, gadolinium (GdCl_3_) protects the myocardium against infarction following ischemia and reperfusion. Neutrophils and macrophages are the main leukocytes responsible for infarct expansion after reperfusion. GdCl_3 _interferes with macrophage and neutrophil function in the liver by decreasing macrophage secretion of inflammatory cytokines and neutrophil infiltration. We hypothesized that GdCl_3 _protects against ischemia and reperfusion injury by decreasing inflammation. We determined the impact of GdCl_3 _treatment for reperfusion injury on 1) circulating monoctye and neutrophil counts, 2) secretion of inflammatory cytokines, and 3) influx of monocytes and neutrophils into the myocardium.

**Methods:**

Rats (n = 3-6/gp) were treated with saline or GdCl_3 _(20 μmol/kg) 15 min prior to a 30 min period of regional ischemia and 120 min reperfusion. Sham rats were not subject to ischemia. Blood was collected either after 30 min ischemia or 120 min reperfusion and hearts were harvested at 120 min reperfusion for tissue analysis. Blood was analyzed for leukocytes counts and cytokines. Tissue was analyzed for cytokines and markers of neutrophil and monocyte infiltration by measuring myeloperoxidase (MPO) and α-naphthyl acetate esterase (ANAE).

**Results:**

GdCl_3 _did not affect the number of circulating neutrophils prior to ischemia. Two hours reperfusion resulted in a 2- and 3- fold increase in circulating monocytes and neutrophils, respectively. GdCl_3 _decreased the number of circulating monocytes and neutrophils during reperfusion to levels below those present prior to ischemia. Furthermore, after 120 min of reperfusion, GdCl_3 _decreased ANAE and MPO activity in the myocardium by 1.9-fold and 6.5-fold respectively. GdCl_3 _decreased MPO activity to levels below those measured in the Sham group. Serum levels of the major neutrophil chemoattractant cytokine, IL-8 were increased from pre-ischemic levels during ischemia and reperfusion in both control and GdCl_3 _treated rats. Likewise, IL-8 levels increased throughout the 3 hour time period in the Sham group. There was no difference in IL-8 detected in the myocardium after 120 min reperfusion between groups. In contrast, after 120 min reperfusion GdCl_3 _decreased the myocardial tissue levels of macrophage secreted cytokines, GM-CSF and IL-1.

**Conclusion:**

GdCl_3 _treatment prior to ischemia and reperfusion injury decreased circulating monocytes and neutrophils, macrophage secreted cytokines, and leukocyte infiltration into injured myocardium. These results suggest GdCl_3 _decreased monoctye and neutrophil migration and activation and may be a novel treatment for inflammation during ischemia and reperfusion.

## Background

The lanthanide cation, gadolinium (GdCl_3_) protects the myocardium against infarction following ischemia and reperfusion (IR) *in vivo *[1], although this preconditioning is not observed in a buffer perfused, isolated heart model of acute reperfusion injury (unpublished observation). This discrepancy suggests that GdCl_3_-induced cardioprotection is dependent upon factors found only *in vivo*, such as blood cells, proteins or hormones among others.

Inflammatory cells are important in the pathophysiological response to injury associated with IR. While crucial to healing, the influx of inflammatory cells, specifically macrophages and neutrophils, results in tissue injury beyond that caused by ischemia alone. Many studies have focused on the acute myocardial inflammatory reaction as a mediator of ischemia-reperfusion injury [2]. Monocytes and other leukocytes infiltrate the area at risk soon after the onset of ischemia. Activated macrophages secrete cytokines that promote tissue damage and recruit neutrophils [3]. Accordingly, the influx of neutrophils into ischemic tissue increases tissue necrosis by releasing proteolytic enzymes and reactive oxygen species and expands the zone of infarction [4].

Strategies aimed at reducing the levels of inflammatory cytokines [5] or the infiltration of leukocytes [6] attenuate myocardial damage associated with reperfusion. Evidence suggests that GdCl_3 _interferes with macrophage and neutrophil function in the liver by decreasing macrophage secretion of inflammatory cytokines and toxic oxygen radicals [7] and by inhibiting neutrophil infiltration [8]. The role GdCl_3 _plays in monocyte and neutrophil infiltration during myocardial ischemia and reperfusion is unknown. Accordingly, this study tests the hypothesis that GdCl_3 _modulates leukocyte function either directly by interfering with migration or indirectly by decreasing the generation of inflammatory cytokines and chemokines, thereby decreasing the signal that triggers leukocytes to infiltrate into the injured tissue.

## Methods

Male Sprague Dawley rats at 8 weeks of age (250-300 g) were used in this study and received humane care in compliance with the "Guide for the Care and Use of Laboratory Animals" published by the US National Institutes of Health (NIH Publication No. 85-23, revised 1996). This project was granted approval by the local IACUC review board.

### Instrumentation, ischemia-reperfusion protocol and GdCl_3 _treatment

Rats were anesthetized with 20-40 mg/kg intraperitoneal sodium pentobarbital. The right jugular vein was cannulated for delivery of saline solution. A catheter was inserted in the left femoral artery to measure both blood pressure and heart rate and to withdraw blood. Pressure and rate measurements were monitored using a Gould PE50 or PE23 pressure transducer connected to a Grass model 7 polygraph. The rats were intubated with a 14-gauge catheter and ventilated at 38-45 breaths/min (Harvard Apparatus, model 683; South Natick, MA) with supplemental oxygen. Atelectasis was prevented by maintaining a positive end-expiratory pressure of 5-10 mm H_2_O. Arterial pH, pCO2 and pO2 were monitored with an AVL 995 pH/blood gas analyzer, and normal values were maintained by adjusting respiratory rate, tidal volume and/or oxygen flow. Body temperature was maintained between 35 and 37°C using a heating pad.

A left thoracotomy was performed, the pericardium was opened and the left coronary artery was identified. A ligature (6-0 Prolene) was passed around the proximal segment of the left coronary artery, and the ends of the suture were threaded through a propylene tube to form a snare. Regional left ventricular ischemia was induced by tightening the snare for 30 min. Coronary artery occlusion was confirmed by epicardial cyanosis and a decrease in blood pressure. Reperfusion was achieved by releasing the snare and was confirmed by a marked hyperemic response of the myocardium. The heart was reperfused for 120 min then excised and assessed for extent of tissue injury.

**Figure 1:**
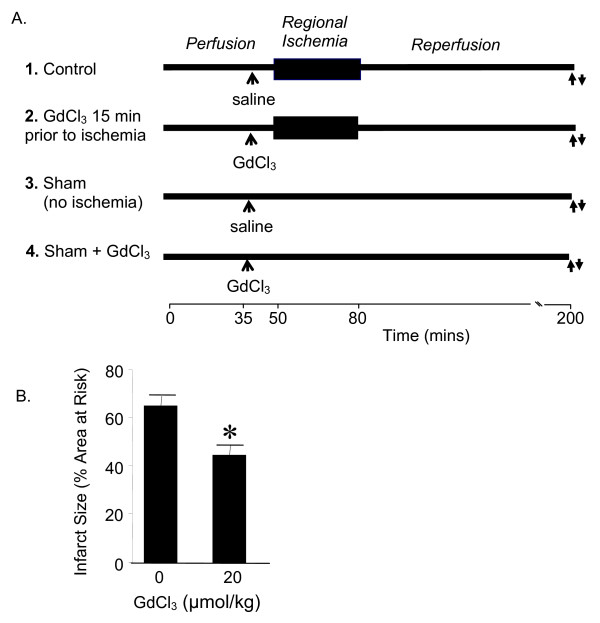
***In vivo *rat model of ischemia and reperfusion injury**. **A) **Rats were treated with either saline or GdCl_3 _(20 μmol/kg) 15 minutes prior to a 30 minute period of regional ischemia and 2 hours reperfusion.↑ Blood collection.↓ Harvest the free wall of the left ventricle. **B) **Measurement of infarct size as a percentage area at risk using this protocol.

Gadolinium chloride hexahydrate (20 μmol/kg dissolved in 0.9% NaCl solution; Sigma, Milwaukee, WI) was given as an intravenous (i.v.) bolus 15 min before inducing myocardial ischemia [1]. Experimental groups are shown in Figure 1A and included Sham (no treatment, no ischemia), Sham + GdCl_3 _treated (treated, no ischemia), Control (no treatment but subject to ischemia and reperfusion) and GdCl_3 _(treated and subject to ischemia and reperfusion) groups. Infarct size was also measured using this protocol and serves as a positive control for this study (Figure 1B).

### Myocardial Tissue Myeloperoxidase Activity Assay

Myeloperoxidase (MPO) activity was assayed as a measure of neutrophil activity in hearts using a modified protocol [9]. The heart was homogenized in 50 mM potassium phosphate buffer (pH 6.0) and centrifuged. The pellet was washed twice in 5 mM potassium phosphate buffer. After washing, the pellets were resuspended in extraction buffer (50 mM potassium phosphate buffer (pH 6.0) containing 0.5% hexadecyltrimethyl ammoniumbromide), followed by three rounds of freeze-thawing. The suspension was incubated at 4°C for 20 minutes and then centrifuged at 13,000 rpm at 4°C for 15 minutes. The supernatant (100 μL) was mixed with 100 μL of reaction buffer (50 mM potassium phosphate buffer (pH 6.0) containing 0.6 mg/mL O-dianisidine dihydrochloride and 0.03% hydrogen peroxide). Absorbance was measured at 450 nm after 5 minutes of incubation. After normalization for protein concentration, the MPO content was expressed as units of MPO activity per milligram of protein.

### Myocardial Tissue α-Naphthyl Acetate Esterase Assay

The activity of α-naphthyl acetate esterase (ANAE), a marker enzyme of monocytes and macrophages was detected using a previous published protocol [10]. In brief, frozen specimens from Sham, Sham + GdCl_3_, Control and GdCl_3 _groups were separately homogenized in ice-cold 0.25 mol/L sucrose (1:5; weight to volume) for 2 × 5 seconds. The samples were centrifuged at 10,000 *g *for 10 min at 4°C. The supernatant was further sonicated for 90 seconds in ice, centrifuged at 105,000 *g *for 90 minutes at 4°C, and assayed for protein. An equivalent of 25 mg of supernatant protein for each sample and 5 mL of 200 mmol/L α-naphthyl acetate (Sigma-Aldrich, St. Louis, MO), dissolved in 95% ethanol to give a final concentration of 0.5 mmol/L, was added to a final volume of 2 mL of saline solution. Blanks received no substrate. After 10 minutes of incubation at 37°C, the reaction was stopped by adding 116 mL of 12.5% w/v sodium dodecyl sulfate solution. Subsequently, 5 mL of 200 mmol/L α-naphthyl acetate was added to the blanks. Finally, 30 mL of fast red solution (10 mg/mL distilled water; Fast Red B, Sigma-Aldrich, St. Louis, MO) was added to the sample, followed by an incubation period of 15 minutes at room temperature. Optical density absorption at 490 nm was used to estimate the metabolism of α-naphthyl acetate to α-naphthol. Alpha-naphthyl acetate esterase activity was estimated as absorption at 490 nm per 25 mg of protein.

### Cytokine Assays

Blood was collected either 30 min after ischemia or 120 min after reperfusion. Plasma was separated by centrifuging the sample at 14,000 rpm × 10 min at 4°C and frozen at -80°C until analysis. Left ventricular free wall tissue homogenates were processed and quantiated using methods described previously [11]. Interleukin (IL)-8 levels were determined using an IL-8 (Rat cytokine-induced neutrophil chemoattractant (CINC)-1) enzyme-linked immunoassay kit from R&D Systems (Minneapolis, MN) according to the manufacture's instructions. CINC-2, CINC-3, granulocyte monocyte colony stimulating factor (GM-CSF), Interferon (INF)-γ, IL-1α, IL-1β, IL-4, IL-6, IL-10, Monocyte chemotactic protein (MCP)-1, Macrophage inflammatory protein (MIP)-3A, and Tumor necrosis factor (TNF)- α tissue levels were determined using RayBio^® ^Rat Cytokine Antibody Array 1 (Norcross, GA) according to the manufacture's instructions. Each dot on the immunoblot representing a cytokine was quantitated using ImageJ 1.37v software.

### Statistical Analysis

Data are reported as mean ± SEM. Statistical analyses were performed by the Student's t test for paired values and a one-way Analysis of Variance (ANOVA) for differences between treatment groups. If significant, a Newman-Keuls multiple comparison test was used to perform pair wise comparisons. Data were considered significant at a p < 0.05. Statistics were performed using WINKS SDA Software (Texasoft, Cedar Hill, TX).

## Results

### GdCl_3 _Attenuates IR-induced Increases in Circulating Monocytes and Neutrophils

**Figure 2:**
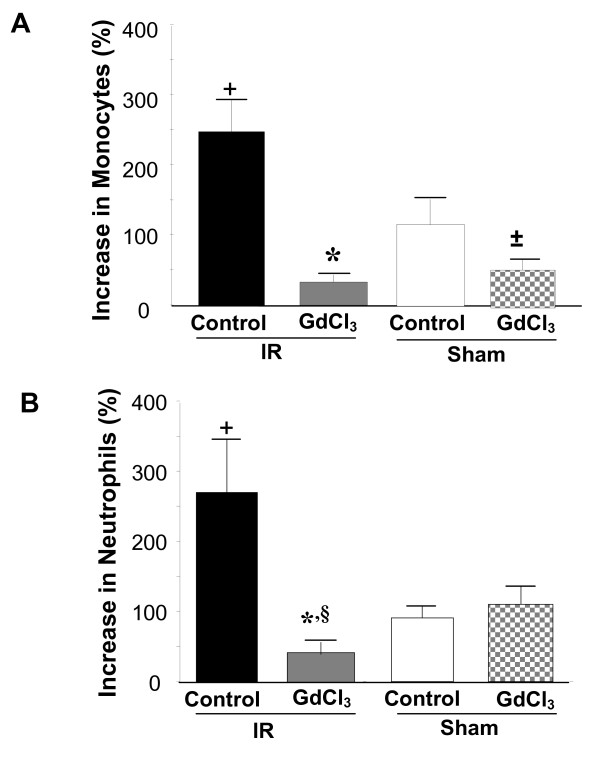
**Gadolinium chloride decreases circulating monocytes and neutrophils following ischemia and reperfusion**. Rats were treated with either saline or GdCl_3 _(20 μmol/kg) 15 minutes prior to a Sham procedure or a 30 minute period of regional ischemia and 2 hours reperfusion. **A) **Increase in monocytes. **B) **Increase in neutrophils. Data mean ± SD, n = 3-6/gp, + = p < 0.05, Sham control vs. Ischemia-Reperfusion (IR) control,* = p < 0.05, IR Control vs. IR GdCl_3_, ± = p < 0.05, Sham Control vs. Sham GdCl_3_, § = p < 0.05, IR GdCl_3_l vs. Sham GdCl_3_

We first determined whether GdCl_3 _decreases circulating monocytes and neutrophils following ischemia and reperfusion. We have previously shown that the optimal cardioprotective dose of GdCl_3 _was 20 μmol/kg (Figure 1B) [1]. Therefore, we used this dose for the experiments in this study. Rats were treated with either saline or GdCl_3 _15 minutes prior to a 30 minute period of regional ischemia followed by 120 min reperfusion. A complete blood count and differential was performed after 120 min reperfusion. GdCl_3 _did not affect the number of circulating monocytes or neutrophils prior to ischemia. Two hours reperfusion resulted in a 2-fold increase in circulating monocytes and a 3-fold increase in circulating neutrophils. GdCl_3 _decreased the number of circulating monocytes (Figure 2A) and neutrophils (Figure 2B) during reperfusion to levels below those present prior to ischemia. In addition, GdCl_3 _given to the Sham group also decreased the number of circulating monocytes, but not neutrophils when compared to the untreated Sham group.

### Macrophage Infiltration in Myocardium

**Figure 3:**
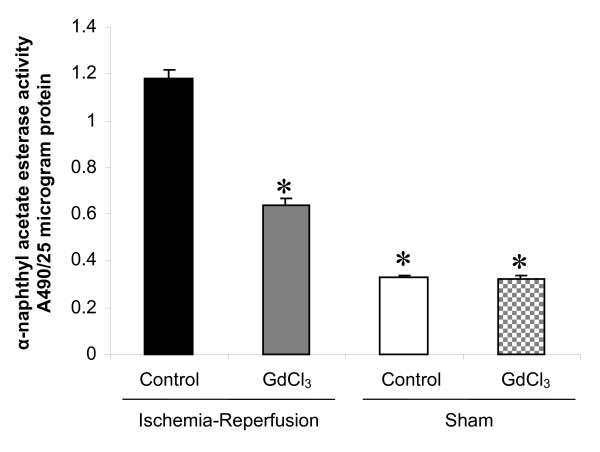
**Gadolinium chloride decreases Alpha naphthyl acetate esterase activity in the reperfused myocardium**. Rats were either treated with vehicle or GdCl_3 _either before 30 min ischemia and 120 min reperfusion or a Sham procedure. Alpha naphthyl acetate esterase activity was measured after 120 min reperfusion in IR Control and IR + GdCl_3 _groups and at a comparable time point in Sham Control and Sham + GdCl_3 _groups. Data is mean ± SD, n = 6/group, * = p < 0.05 vs. IR control.

Using α-naphthyl acetate esterase (ANAE) content as an indicator of myocardial tissue monocyte/macrophage infiltration, ischemia and reperfusion increased ANAE activity by 3.6-fold in the IR control group when compared the Sham control group (Figure 3). GdCl_3 _partially reversed increase in ANAE activity after ischemia and reperfusion injury by 1.9-fold but this was still above Sham values. GdCl_3 _did not change ANAE activity in the Sham-GdCl_3 _group when compared to the Sham control group.

### Neutrophil Infiltration in Myocardium

**Figure 4:**
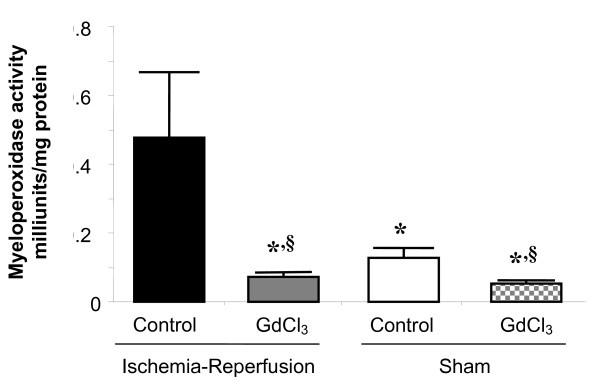
**Gadolinium chloride decreases myocardial tissue myeloperoxidase activity**. Rats were either treated with vehicle or GdCl_3 _either before 30 min ischemia and 120 min reperfusion or a Sham procedure. Myeloperoxidase activity was measured after 120 min reperfusion in IR Control and IR + GdCl_3 _groups and at a comparable time point in Sham and Sham + GdCl_3 _groups. Data is mean ± SD, n = 3/group, * = p < 0.05 vs. IR control; § = p < 0.05 vs. Sham control.

Using myeloperoxidase (MPO) content as an indicator of myocardial tissue neutrophil infiltration, ischemia and reperfusion increased MPO activity by 3.8-fold in control hearts when compared to a Sham procedure (Figure 4). GdCl_3 _not only reversed this increase in MPO activity after ischemia and reperfusion injury by 6.5-fold when compared to IR control but also decreased MPO activity below Sham control values by 1.7-fold. In the Sham groups, GdCl_3 _decreased MPO activity 2.4-fold.

### GdCl_3 _Modulation of Cytokine Levels

**Figure 5:**
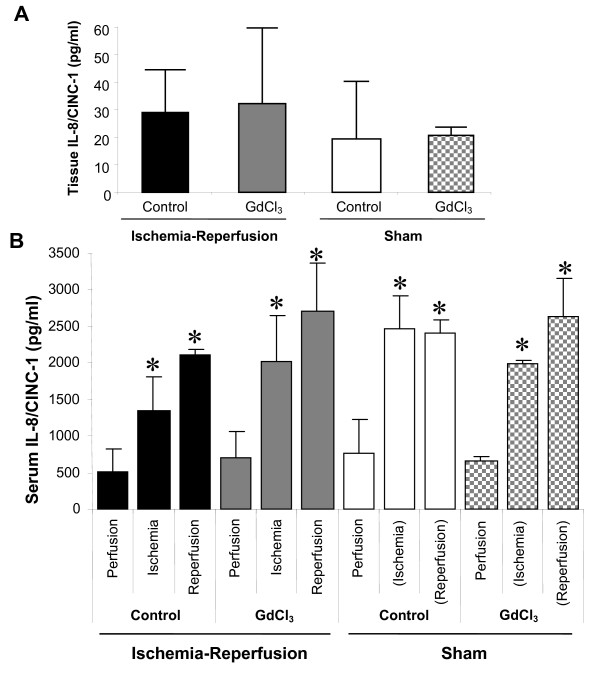
**Gadolinium chloride does not decrease IL-8/CINC-1 production in the myocardium or serum during ischemia and reperfusion**. Rats were either treated with vehicle or GdCl_3 _either before 30 min ischemia and 120 min reperfusion or a Sham procedure. (Ischemia) and (Reperfusion) in the Sham groups is equal to the time period that correlates with Ischemia and Reperfusion in the Control and GdCl_3 _groups. IL-8/CINC-1 was measured from the serum at 30 min ischemia and 120 min reperfusion and from the tissue at 120 min reperfusion. **A) **Quantitation of tissue IL-8/CINC-1 **B) **Quatitation of serum IL-8/CINC-1. Data mean ± SD, n = 3-4/gp, * = p < 0.05 vs. perfusion. No significant difference between IR vs. Sham groups or Control vs. GdCl_3 _groups.

Inflammatory cytokines were then measure from either serum or myocardial tissue at the end of ischemia or reperfusion periods. There was no difference in IL-8/CINC-1 levels in the serum or the tissue when measured after 30 min ischemia (serum) or 120 min reperfusion (serum and tissue) (Figure 5A and 5B). Interestingly, IL-8/CINC-1 serum levels increased throughout the study in all groups when compared to the respective baseline levels.

**Figure 6:**
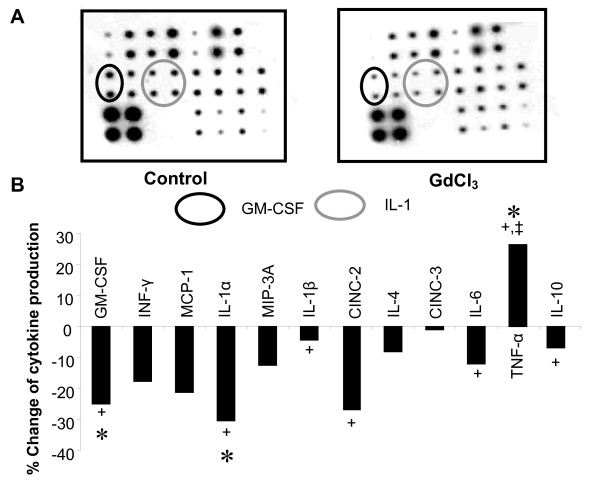
**Gadolinium chloride decreases macrophage secreted cytokines after ischemia and reperfusion in rats**. **(A) **Representative blots from cytokine arrays from control hearts vs. GdCl_3_-treated hearts. The GM-CSF and IL-1 dots are indicated. Dots were quantitated and normalized to the positive control dots (lower left 4 dots) and results displayed above **(B)**. Data mean ± SD, n = 3-4/gp, * = p < 0.05, GdCl_3 _vs. control, += macrophage secreted cytokines, ‡ = cardiomyocyte secreted cytokines.

A macroarray was performed using homogenized tissue after 120 min reperfusion to look for the tissue levels of CINC-2, CINC-3, GM-CSF, INF-γ, IL-1α, IL-1β, IL-4, IL-6, IL-10, MCP-1, MIP-3A, and TNF-α. GdCl3 treatment did not reduce the major monocyte chemoattactant, MCP-1. However, GdCl_3_-treatment did reduce the macrophage secreted cytokines such as GM-CSF and IL-1 after 120 min reperfusion (Figure 6). In addition, an increase in TNF-α was observed in the GdCl_3 _groups after 120 min reperfusion.

## Discussion

The important finding in this study is that a single treatment of GdCl_3 _prior to ischemia decreased the numbers of circulating monocytes and neutrophils following reperfusion and reduced infiltration of these leukocytes into the injured myocardium. In addition, GdCl_3 _decreased production of cytokines that are typically secreted by monocytes. These finding are associated with GdCl_3_-mediated reduction in infarct size after ischemia and reperfusion [1].

The early phase of myocardial infarction is associated with tissue infiltration of circulating leukocytes in response to chemotactic factors [12]. As the leukocytes infiltration the tissue and become activated, they release more cytokines thereby recruiting further leukocytes to the injured area. Activated leukocytes within the tissue cause further tissue injury by releasing reactive oxygen species and proteases and cause capillary plugging leading further tissue hypoxia. Inhibiting neutrophil infiltration using anti-neutrophil antibodies [13], neutrophil depleting antimetabolites [14] or neutrophil filters [15] have been successful in limiting myocardial infarct size. We have shown that GdCl_3 _is also a potent monocyte and neutrophil depleting compound as it prevents these leukocytes from circulating and infiltrating into the myocardium.

Gadolinium is known to modulate inflammatory responses by liver macrophages (Kupfer cells) [7,16]. Gadolinium causes a 70% reduction in the phagocytosis of radiolabeled bacteria [17], and GdCl_3 _has been shown to prevent the release of both inflammatory cytokines and toxic oxygen radicals such as superoxide anion produced by activated Kupffer cells [18,19]. Gadolinium also upregulates secretion of TNF-α from Kupfer cells in response to endotoxemia.

In the present study, GdCl_3 _treatment pre-ischemia was associated with attenuation of the IR-induced increases in circulating monocytes and neutrophils, and it decreased circulating monocytes in animals not exposed to IR. The selective action of GdCl_3 _on monocytes is based on the fact that this compound is readily dissolved in normal saline; however, when it is injected into the bloodstream, it rapidly aggregates into relatively large colloidal particles at neutral pH. The particles of GdCl_3 _are taken up exclusively by circulating phagocytic mononuclear cells of a monocyte lineage (CD11b+, CD13+, and CD14+) [20,21]. Once inside the cell and after exceeding the threshold concentration, GdCl_3 _causes cell apoptosis [22]. The GdCl_3 _aggragates are not expected to cross the endothelial barrier and thus would not be taken up by resident tissue phagocytic cells.

This decrease in circulating leukocytes was not associated with any changes in the levels of the major monocyte chemoattractants, MCP-1 and the neutrophil chemoattractants IL-8/CINC-1, CINC-2, and CINC-3 suggesting that GdCl_3 _modulates leukocyte trafficking to the heart via some other, as yet undefined mechanism following myocardial IR. Interleukin-8/CINC-1 serum levels increased over baseline levels during ischemia and reperfusion. Interestingly, there was a similar increase in serum levels at the corresponding time points in the Sham group. Although the Sham animals do not undergo LAD occlusion, they still receive a tracheostomy and a thoracotomy. These two invasive surgical procedures may be enough to induce an inflammatory response in these rats.

Gadolinium triggered an increase of TNF-α levels in the myocardium after 120 min reperfusion (Figure 6B). TNF-α is a potent cytokine which is elevated in a variety of inflammatory conditions. Endogenous TNF-α has been correlated with the deterioration of myocardial performance, while blockade of TNF-α with a neutralizing antibody preserves myocardial function after IR [23]. Somewhat paradoxically, exogenously added TNF-α is protective during ischemia and reperfusion through mechanisms which involve the JAK/STAT3 pathway, K_ATP _channels and the mitochondrial permeability transition pore [24,25]. This protective effect of TNF-α may be related to our previous data, which showed that GdCl_3_-induced cardioprotection is dependent upon the activation of JAK-STAT3 and K_ATP _channels [1].

We have previously shown that GdCl_3 _given 24-72 hours prior to the index ischemic event is also able to attenuate reperfusion injury as determined by infarct size [1]. Interesting, in that study even though GdCl_3 _was intravenously administered to the rats *in vivo*, infarct size was determined in isolated buffer perfused hearts subject to IR injury. Since the isolated heart model of IR does not have circulating blood components, the infarcts are not likely solely caused by the inflammatory response. However, it still could be possible that GdCl_3_-mediated up-regulation of TNF-α in the myocardium could be part of the mechanism of GdCl_3_-mediated delayed cardioprotection. TNF-α was not measured in the delayed cardioprotection studies but this hypothesis forms the basis of further studies.

Gadolinium also attenuated IR-induced expression of the macrophage-secreted cytokines GM-CSF and IL-1 in this study, suggesting that attracted monocytes may not be differentiating into activated macrophages. Treatment with GdCl_3 _also did not significantly alter levels of INF-γ and MIP-3 which are activators of macrophage function. In support, we observed a decrease in ANAE activity in the myocardium which suggests decreased infiltration and activation of monocytes into the myocardium. Similarly, we observed decreased MPO activity in the myocardial tissue which suggests a decrease of neutrophil infiltration into the myocardium although the levels of circulating and tissue IL-8/CINC-1 are not modified by GdCl_3 _treatment. This suggests that GdCl_3 _may be inhibiting leukocyte chemotaxis and in essence "paralyzing" the monocytes and neutrophils at their storage locations. Swirski et al. have recently identified the splenic subcapsular red pulp as the reservoir for monocytes [26]. From their splenic reservoirs, the monocytes are deployed to inflammatory sites such as the infarcted myocardium [26]. The reservoir of neutrophils is yet to be verified but the neutrophils are thought to reside in the post-capillary venule sites in the periphery such as the bone marrow [27]. Although previous studies have shown that GdCl_3 _does not affect the migration of neutrophils [28], inhibition of monocyte migration has not been reported.

One limitation of this study is that we did not study the *in vitro *migration of isolated monocytes or neutrophils treated with GdCl_3_. However, the decrease in monocytes and neutrophils into the circulation after GdCl_3 _treatment indicates that the leukocytes are either not migrating from their reservoirs or are being scavenged prior to the 120 min reperfusion time point.

In summary, a single dose of GdCl_3 _when given prior to ischemia and reperfusion decreased circulating monocytes and neutrophils as well as decreased the infiltration of these leukocytes into the myocardium. Furthermore, this was not associated with a change in tissue levels of the major monocyte chemoattractant MCP-1 or neutrophil chemoattractant IL-8/CINC-1 but was associated with an up-regulation of TNF-α.

## List of abbreviations

(GdCl_3_): Gadolinium; (IR): Ischemia and Reperfusion; (IL): Interleukin; (CINC): cytokine-induced neutrophil chemoattractant; (GM-CSF): monocyte colony stimulating factor; (INF): Interferon; (MCP): monocyte chemotactic protein; (MIP): macrophage inflammatory protein; (TNF): tumor necrosis factor; (ANOVA): Analysis of Variance.

## Competing interests

The authors declare that they have no competing interests.

## Authors' contributions

JLS conceived the study, designed the experiments, performed the cytokine assays, data analysis, and prepared the manuscript. KVR performed the MPO and ANAE assays. AH performed the rat studies needed. ACN contributed to the development of the study and revising the manuscript critically for important intellectual content. JEB supervised the experiments and oversaw manuscript construction with JLS. All authors read and approved the final manuscript.

## References

[B1] Nicolosi AC Strande JL Hsu A Fu X Su J Gross GJ Baker JE Gadolinium limits myocardial infarction in the rat: dose-response, temporal relations and mechanisms J Mol Cell Cardiol 2008 44 345 351 10.1016/j.yjmcc.2007.11.002 18083188 PMC2322938

[B2] Zahler S Massoudy P Hartl H Hahnel C Meisner H Becker BF Acute cardiac inflammatory responses to postischemic reperfusion during cardiopulmonary bypass Cardiovasc Res 1999 41 722 730 10.1016/S0008-6363(98)00229-6 10435044

[B3] Colletti LM Kunkel SL Walz A Burdick MD Kunkel RG Wilke CA Strieter RM Chemokine expression during hepatic ischemia/reperfusion-induced lung injury in the rat. The role of epithelial neutrophil activating protein J Clin Invest 1995 95 134 141 10.1172/JCI117630 7814607 PMC295389

[B4] Frangogiannis NG Smith CW Entman ML The inflammatory response in myocardial infarction Cardiovasc Res 2002 53 31 47 10.1016/S0008-6363(01)00434-5 11744011

[B5] Crawford MH Grover FL Kolb WP McMahan CA O'Rourke RA McManus LM Pinckard RN Complement and neutrophil activation in the pathogenesis of ischemic myocardial injury Circulation 1988 78 1449 1458 3191598 10.1161/01.cir.78.6.1449

[B6] Altavilla D Squadrito F Ioculano M Canale P Campo GM Zingarelli B Caputi AP E-selectin in the pathogenesis of experimental myocardial ischemia-reperfusion injury Eur J Pharmacol 1994 270 45 51 7512508 10.1016/0926-6917(94)90079-5

[B7] Iimuro Y Yamamoto M Kohno H Itakura J Fujii H Matsumoto Y Blockade of liver macrophages by gadolinium chloride reduces lethality in endotoxemic rats--analysis of mechanisms of lethality in endotoxemia J Leukoc Biol 1994 55 723 728 8195698 10.1002/jlb.55.6.723

[B8] Chen YX Sato M Kawachi K Abe Y Neutrophil-mediated liver injury during hepatic ischemia-reperfusion in rats Hepatobiliary Pancreat Dis Int 2006 5 436 442 16911946

[B9] Hillegass LM Griswold DE Brickson B Albrightson-Winslow C Assessment of myeloperoxidase activity in whole rat kidney J Pharmacol Methods 1990 24 285 295 10.1016/0160-5402(90)90013-B 1963456

[B10] Formigli L Manneschi LI Nediani C Marcelli E Fratini G Zecchi Orlandini S Perna AM Are macrophages involved in early myocardial reperfusion injury? The Annals of Thoracic Surgery 2001 71 1596 1602 10.1016/S0003-4975(01)02400-6 11383806

[B11] Strande JL Hsu A Su J Fu X Gross GJ Baker JE Inhibiting protease-activated receptor 4 limits myocardial ischemia/reperfusion injury in rat hearts by unmasking adenosine signaling J Pharmacol Exp Ther 2008 324 1045 1054 10.1124/jpet.107.133595 18055876 PMC2935083

[B12] Bosnjak JJ Terata K Miura H Sato A Nicolosi AC McDonald M Manthei SA Saito T Hatoum OA Gutterman DD Mechanism of thrombin-induced vasodilation in human coronary arterioles Am J Physiol Heart Circ Physiol 2003 284 H1080 1086 12595282 10.1152/ajpheart.00465.2002

[B13] Romson JL Hook BG Kunkel SL Abrams GD Schork MA Lucchesi BR Reduction of the extent of ischemic myocardial injury by neutrophil depletion in the dog Circulation 1983 67 1016 1023 6831665 10.1161/01.cir.67.5.1016

[B14] Mullane KM Read N Salmon JA Moncada S Role of leukocytes in acute myocardial infarction in anesthetized dogs: relationship to myocardial salvage by anti-inflammatory drugs J Pharmacol Exp Ther 1984 228 510 522 6420544

[B15] Engler RL Dahlgren MD Morris DD Peterson MA Schmid-Schonbein GW Role of leukocytes in response to acute myocardial ischemia and reflow in dogs Am J Physiol 1986 251 H314 323 3740286 10.1152/ajpheart.1986.251.2.H314

[B16] Rai RM Yang SQ McClain C Karp CL Klein AS Diehl AM Kupffer cell depletion by gadolinium chloride enhances liver regeneration after partial hepatectomy in rats Am J Physiol 1996 270 G909 918 8764196 10.1152/ajpgi.1996.270.6.G909

[B17] Husztik E Lazar G Parducz A Electron microscopic study of Kupffer-cell phagocytosis blockade induced by gadolinium chloride Br J Exp Pathol 1980 61 624 630 7459256 PMC2041618

[B18] Vega VL Maldonado M Mardones L Schulz B Manriquez V Vivaldi E Roa J Ward PH Role of Kupffer cells and PMN leukocytes in hepatic and systemic oxidative stress in rats subjected to tourniquet shock Shock 1999 11 403 410 10454829

[B19] Kono H Fujii H Matsuda M Yamamoto M Matsumoto Y Gadolinium chloride prevents mortality in hepatectomized rats given endotoxin J Surg Res 2001 96 204 210 10.1006/jsre.2001.6099 11266274

[B20] Wasserman AJ Monticello TM Feldman RS Gitlitz PH Durham SK Utilization of Electron Probe Microanalysis in Gadolinium-Treated Mice Toxicol Pathol 1996 24 588 594 10.1177/019262339602400508 8923680

[B21] Frid MG Brunetti JA Burke DL Carpenter TC Davie NJ Reeves JT Roedersheimer MT van Rooijen N Stenmark KR Hypoxia-Induced Pulmonary Vascular Remodeling Requires Recruitment of Circulating Mesenchymal Precursors of a Monocyte/Macrophage Lineage Am J Pathol 2006 168 659 669 10.2353/ajpath.2006.050599 16436679 PMC1606508

[B22] Singh B Pearce JW Gamage LN Janardhan K Caldwell S Depletion of pulmonary intravascular macrophages inhibits acute lung inflammation Am J Physiol Lung Cell Mol Physiol 2004 286 L363 372 10.1152/ajplung.00003.2003 14565942

[B23] Gurevitch J Frolkis I Yuhas Y Lifschitz-Mercer B Berger E Paz Y Matsa M Kramer A Mohr R Anti-tumor necrosis factor-alpha improves myocardial recovery after ischemia and reperfusion J Am Coll Cardiol 1997 30 1554 1561 10.1016/S0735-1097(97)00328-8 9362416

[B24] Gao Q Zhang S-Z Cao C-M Bruce IC Xia Q The mitochondrial permeability transition pore and the Ca2+-activated K+ channel contribute to the cardioprotection conferred by tumor necrosis factor-[alpha] Cytokine 2005 32 199 205 10.1016/j.cyto.2005.09.008 16260145

[B25] Lecour S Suleman N Deuchar GA Somers S Lacerda L Huisamen B Opie LH Pharmacological preconditioning with tumor necrosis factor-alpha activates signal transducer and activator of transcription-3 at reperfusion without involving classic prosurvival kinases (Akt and extracellular signal-regulated kinase) Circulation 2005 112 3911 3918 10.1161/CIRCULATIONAHA.105.581058 16344382

[B26] Swirski FK Nahrendorf M Etzrodt M Wildgruber M Cortez-Retamozo V Panizzi P Figueiredo JL Kohler RH Chudnovskiy A Waterman P Identification of splenic reservoir monocytes and their deployment to inflammatory sites Science 2009 325 612 616 10.1126/science.1175202 19644120 PMC2803111

[B27] Babior BM Golde DW Beutler E, Coller BS, Lichtman MA, Kipps TJ, Seligsohn U Production, distribution, and fate of neutrophils Williams Hematology 2001 New York: McGraw-Hill 753 759

[B28] Elferink JGR de Koster BM Inhibition of interleukin-8-activated human neutrophil chemotaxis by thapsigargin in a calcium-and cyclic AMP-dependent way Biochemical Pharmacology 2000 59 369 375 10.1016/S0006-2952(99)00342-1 10644044

